# Pityriasis Lichenoides Chronica Associated with Herpes Simplex Virus Type 2

**DOI:** 10.1155/2012/737428

**Published:** 2012-12-02

**Authors:** Antonio Javier González Rodríguez, Encarnación Montesinos Villaescusa, Esperanza Jordá Cuevas

**Affiliations:** Departments of Dermatology, Hospital Clínico of Valencia, University of Valencia, Avenida Blasco Ibáñez 17, 46010 Valencia, Spain

## Abstract

*Introduction*. Pityriasis lichenoides is a rare, acquired spectrum of skin conditions of an unknown etiology. *Case Report*. A 28-year-old man presented with recurrent outbreaks of herpes simplex virus associated with the onset of red-to-brown maculopapules located predominantly in trunk in each recurrence. Positive serologies to herpes simplex virus type 2 were detected. Histopathological examination of one of the lesions was consistent with a diagnosis of pityriasis lichenoides chronica. *Discussion*. Pityriasis lichenoides is a rare cutaneous entity of an unknown cause which includes different clinical presentations. A number of infectious agents have been implicated based on the clustering of multiple outbreaks and elevated serum titers to specific pathogens (human immunodeficiency virus, cytomegalovirus, Epstein-Barr virus, *Toxoplasma gondii*, and herpes simplex virus). In our patient, resolution of cutaneous lesions coincided with the administration of antiviral drugs and clinical improvement in each genital herpes recurrence. In conclusion, we report a case in which cutaneous lesions of pityriasis lichenoides chronica and a herpes simplex virus-type 2-mediated disease have evolved concomitantly.

## 1. Introduction

Pityriasis lichenoides is a rare, acquired spectrum of skin conditions of an unknown etiology, although infectious agents have been suspected as etiologic factors.

## 2. Case Report

**Figure 1:**
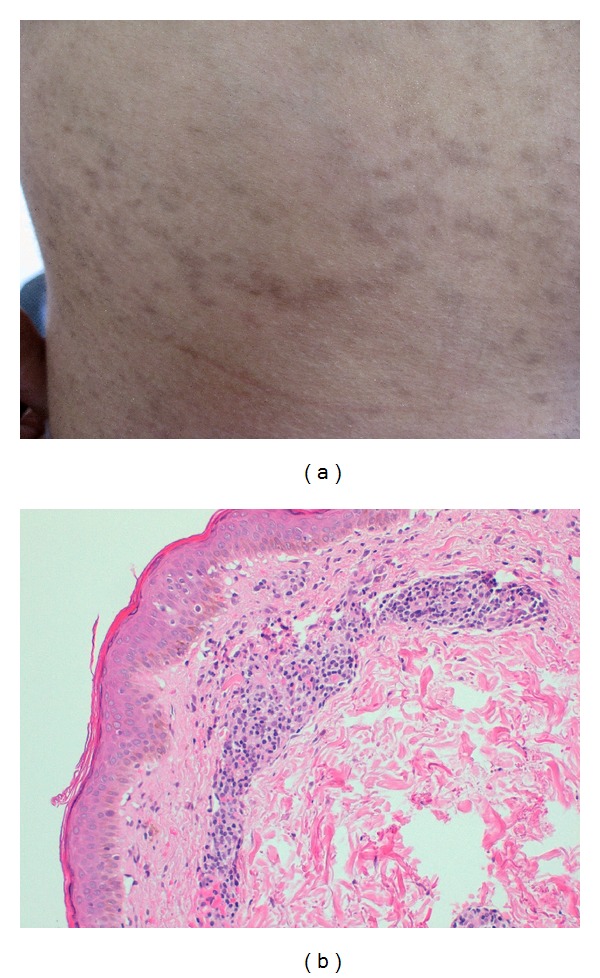
(a) Red-to-brown maculopapules in trunk. (b) Epidermal acanthosisand scanty lymphocytic exocitosis. Perivascular infiltrates of lymphocytes in dermis (haematoxylin and eosin, original magnification ×20).

A 28-year-old man presented with recurrent outbreaks of herpes simplex virus (HSV) type 2 in the form of ulcers on the glans penis associated with the onset of red-to-brown maculopapules located predominantly in trunk in each recurrence. The patient had four recurrences on a two-year followup (Figure 1(a)).

Positive serologies to HSV type 2 were detected in each outbreak (IgM and IgG). He received acyclovir 400 mg five times a day for 5 days, then a prophylactic dose of 400 mg twice a day; however, after discontinuation of antiviral drug, lesions reappeared.

Histopathological examination of one of the lesions showed epidermal acanthosis, scanty lymphocytic exocytosis, without spongiosis, or focal necrosis. Perivascular infiltrates of lymphocytes with occasional eosinophils and erythrocytic extravasation were seen in the dermis (Figure 1(b)). HSV DNA was not detected in the epidermal keratinocytes or lymphocytes in the biopsy specimen. These findings were consistent with a diagnosis of pityriasis lichenoides chronica.

## 3. Discussion

Pityriasis lichenoides (PL) is a rare cutaneous entity of an unknown cause which includes different clinical presentations. It encompasses a spectrum of clinical presentations ranging from acute papular lesions rapidly evolving to form pseudovesicles and central necrosis (pityriasis lichenoides et varioliformis acuta or PLEVA) to small, scaling, benign-appearing papules (pityriasis lichenoides chronica or PLC). PLEVA and PLC are not distinct diseases but are rather different manifestations of the same process. The pathogenic mechanism behind PL is unclear although infectious or drug-related hypersensitivity reactions versus lymphoproliferative disorders are the mainstay theories. A number of infectious agents have been implicated based on the clustering of multiple outbreaks and elevated serum titers to specific pathogens, such as human immunodeficiency virus, hepatitis C virus, cytomegalovirus, Epstein-Barr virus, Toxoplasma gondii, and HSV [1–4], but there has been no consistent finding so far. Recent studies have demonstrated PLEVA to be a benign disorder of activated T-cell lymphocytes. Furthermore, monoclonal rearrangement of the T-cell receptor-gamma (TCR-gamma) genes was repeatedly found both in PLEVA and PLC [5–7].

In our patient, resolution of cutaneous lesions coincided with the administration of antiviral drugs (they remained as residual lesions without scarring) and clinical improvement in each genital herpes recurrence. PLC did not become less severe at successive attacks. Whether this association represents HSV reactivation caused by immune dysfunction related to the skin disease or HSV acting as an antigenic trigger is uncertain.

In conclusion, we report a case in which cutaneous lesions of pityriasis lichenoides chronica and a herpes simplex virus type 2-mediated disease have evolved concomitantly.
